# Classification of lesions inducing acquired cholesteatomas of the middle ear: a didactic suggestion^☆^

**DOI:** 10.1016/j.bjorl.2018.07.001

**Published:** 2018-07-17

**Authors:** Fernando de Andrade Quintanilha Ribeiro

**Affiliations:** Faculdade de Medicina da Santa Casa de São Paulo, São Paulo, SP, Brazil

We consider lesions inducing acquired cholesteatomas as any lesion that has the potential to induce the development of cholesteotoma in the middle ear. Several studies have assessed cholesteatomas regarding their possible causes, their evolution and their histological characteristics and treatments. Several classifications have been made regarding their location.^1^, ^2^ A consensus on the definition and classification of cholesteatomas was carried out by the European Academy of Otology and Neurotology (EAONO) and the Japan Otological Society (JOS), since both Institutions already had previous distinct classifications.^3^, ^4^, ^5^ Basically, middle ear cholesteatomas are classified as congenital and acquired. The acquired cholesteatomas can originate from the “pars tensa”, or the “pars flaccida” or can be indeterminate, usually by the development of retraction pockets. The ones originating from “pars tensa” can be anterior or posterior epitympanic and posterior mesotympanic cholesteatomas. The term “primary” was abolished, and the term “secondary” was restricted to cholesteatomas arising from skin migration through pre-existing perforations of the tympanic membrane, a very uncommon situation.

However, in all the published studies, little is said about the characteristics of their primordial lesions. Cholesteatomas are classified descriptively and when they are already structured: skin migrations through perforations (rarer) and regions of tympanic membrane affected by negative pressure, or retraction pockets without objectively characterizing them regarding the degree of development or prognosis (considering the dynamics of the process). It is necessary to adequately describe an initial lesion, so that it can be easily perceived and recalled, as the conduct of care, the follow-up, the surgical urgency, the prognosis that depend on it.

We know that words sometimes do not adequately represent what we are trying to define. This is not rare when one wants to explain a clinical finding, describe a surgery, or even explain and publish a study. We often are not able to describe what we are really seeing in others. Aiming to allow an easier communication, we often use analogies in anatomy and pathology to construct the desired image. Thus, why not use these resources to make us better understand, and to make everyone capable of adequately visualizing, while using the same standard, what we want to depict in the description of tympanic lesions?

To adequately classify lesions inducing acquired cholesteatomas of the middle ear (negative pressure and retraction pockets), we have used a classification based on well-known domestic utensils, thus requiring no memorization skills. This process aimed to facilitate the use of a guide, especially for students, including the evolution, treatment and prognosis in each case, for their appropriate management. Obviously, treatment choices will vary according to each otolaryngology department guideline.

To consolidate our experience, a small questionnaire was applied to individuals attending our Digital Otology Outpatient Clinic. The project was submitted to the Institution's Ethics Board (2346725) for its approval, after obtaining free and informed consent form from the participants.

Congenital cholesteatomas and secondary cholesteatomas due to skin migration through a pre-existing perforation, rare in our sample, were not evaluated, neither the residual and recurrent cholesteatomas, both post-surgical types.

This classification with a didactic guidance was presented to six otorhinolaryngologists with different degrees of experience, namely, a professor, a fellow, two third-year residents and two second-year residents. They were shown 41 scanned otoscopic images, which they had to classify according to the proposed morphological characteristics. The standard for comparison was defined by the author (Fig. 1).

**Figure 1:**
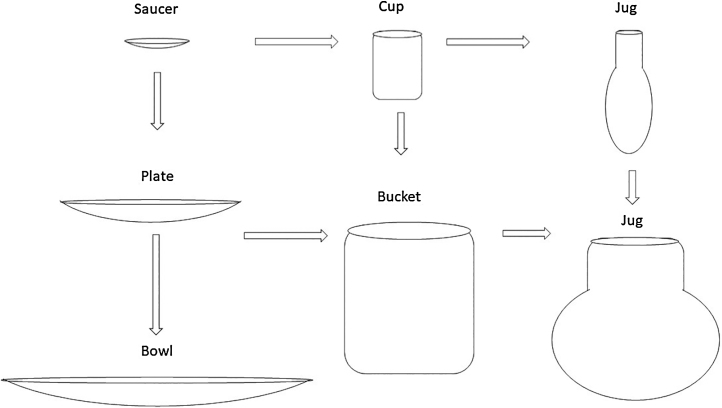
Evolution of the lesions.

## Saucer

Small, shallow and empty – Normal and frequent anatomical finding in the “pars flaccida” region of the membrane, close to the short apophysis of the malleus. Some individuals may have a predisposition for “pars flaccida” cholesteatoma. It is usually bilateral and may appear on the tense side, resembling a small neotympanum area.

Treatment – Expectant, but it should be followed clinically, if it occurs in a patient with Eustachian tube dysfunction, when it can come under the influence of negative pressure and progress to a cholesteatoma, especially if this has already occurred in the contralateral ear.

## Cup

Deeper than the saucer, it does not retain skin or secretion, and can be the evolution of the saucer type in the “pars flaccida” or occur in the “pars tensa” as an initial area under the influence of negative pressure of a small neotympanum.

Treatment – It should not be a condition of concern if it can be reversed by Valsava maneuver. Otherwise, may be submitted to surgery (mechanically reversed). In such cases, a reinforcement is made in the affected membrane zone, using fascia or perichondrium to prevent its evolution to a cholesteatoma.

## Plate

With a wide “mouth” and empty, it can be found in the “pars flaccida”, as the saucer enlargement or in the “pars tensa”, as an initial area under the influence of negative pressure of a large neotympanum.

Treatment – The plate in the “pars flaccida” is the evolution of the saucer and may become a reason of concern due to the possible involvement of the ossicular chain. In the “pars tensa” it is usually reversed by the Politzer or Valsalva maneuver. Otherwise, it should be surgically detached, and reinforced with fascia, perichondrium or even cartilage, to maintain the tympanic membrane semirigid structure. If the treatment does not seem feasible due to the possible rupture in the detachment, it should be treated expectantly, as it does not always develop into a cholesteatoma.

## Bucket

With a “wide” mouth, deeper, but still empty (without skin cells retention), it is the evolution of a plate or cup into the attico-antral region. It may be located in the “pars flaccida” (rarer) or be the partial retraction of the membrane “pars tensa”.

Treatment – Difficult surgical treatment due to the risk of skin rupture and entrapment, which will result in an iatrogenic cholesteatoma. This procedure may be attempted if the lesion is not attached to the ossicular chain, which would usually be partially eroded.

## Bowl

Total retraction of the tympanic membrane, atelectasis or adhesive membrane. It begins with the retraction of the atrophic membrane, which later touches the incus and can erode the latter or adhere to the promontory.

Treatment – Depending on the stage, it can be controlled with tympanotomy with insertion of a ventilation tube, Valsalva or Politzer maneuvers or by strengthening the tympanic membrane with fascia, perichondrium or cartilage. It tends to suffer the influence of a negative pressure into the attic and antrum region, constituting a “pars tensa” cholesteatoma.

## Jug

With a small “mouth” in relation to its “belly”, which is larger, it retains desquamated skin, and usually becomes infected, no longer characterized by a retraction pocket, but actually a cholesteatoma. It can be the evolution of the saucer/cup type in the “pars flaccida” region or retraction in the “pars tensa”. The size of its “belly”, whether deeper or wider, can often be determined only through imaging (CT) or the surgical procedure. It tends to migrate to the attico-antral region and can occupy much of the mastoid process.

Treatment – Surgical. Depending on the size and location, surgery is performed through a canal wall down mastoidectomy (radical) or canal wall up mastoidectomy (tympanomastoidectomy). Whenever possible, the second method should be used in order to preserve the middle ear anatomy and function; but a canal wall up mastoidectomy can be more problematic due to frequent recurrences.

## Undetermined

It shows a large amount of skin migration and infection, and it is not possible to determine the real origin.

Treatment – Surgical, usually through radical or canal wall down mastoidectomy.

This classification can be further described according to the lesion location and characteristics. For instance: *shallow bucket* in the anterior epitympanum region. That is, the retraction of a neotympanum, near the area of the Eustachian tube, of which the bottom shows no skin retention or infection. Or, *bowl* with jug formation under the malleus handle, characterized by membrane atelectasis with a retraction area located in the attic region, with desquamation and secretion, characterizing a cholesteatoma. Or, *saucer* in the “pars flaccida”, tending toward a cup shape, indicating some negative pressure retraction with the tendency to become an attic cholesteatoma.

For each shape (41) presented, the participants classified the lesions according to their morphology, following the proposed classification. Evaluator 1 was a professor, number 2 was a fellow, numbers 3 and 4 were third-year residents (R3) and numbers 5 and 6 were second-year residents (R2). Therefore, we obtained the results regarding the correct/wrong response regarding the aspect of the lesion from the six assessed participants, in comparison with the standard established by the author (in percentage):

**Table:** 

Evaluator	Lesion
ev1	87.8
ev2	97.6
ev3	85.4
ev4	82.9
ev5	90.24
ev6	78.05

Thus, we observed a significantly high percentage of correct responses from all participants, even among the least experienced.

We believe that this classification of conditions that can induce acquired cholesteatomas and the comments on their evolution and management can clarify the concepts of their dynamics and progression, especially for students. It may also be helpful for describing adequately these clinical conditions and findings to another doctor in a medical file or chart for future reevaluations, and most importantly, to understand the characteristics of the initial lesions, which, if not adequately managed, can lead to serious complications and expensive treatment.

This classification and comments do not aim to teach otology, they are just didactic suggestions that have been successfully used with great acceptance in our department.

## Conflicts of interest

The author declares no conflicts of interest.
